# Key Odorants Regulate Food Attraction in *Drosophila melanogaster*

**DOI:** 10.3389/fnbeh.2017.00160

**Published:** 2017-09-05

**Authors:** Thomas Giang, Jianzheng He, Safaa Belaidi, Henrike Scholz

**Affiliations:** Department of Biology, Albertus-Magnus University of Cologne Cologne, Germany

**Keywords:** *Drosophila melanogaster*, food odor, key odorants, Orco, odor background intensity, ethanol

## Abstract

In insects, the search for food is highly dependent on olfactory sensory input. Here, we investigated whether a single key odorant within an odor blend or the complexity of the odor blend influences the attraction of *Drosophila melanogaster* to a food source. A key odorant is defined as an odorant that elicits a difference in the behavioral response when two similar complex odor blends are offered. To validate that the observed behavioral responses were elicited by olfactory stimuli, we used olfactory co-receptor *Orco* mutants. We show that within a food odor blend, ethanol functions as a key odorant. In addition to ethanol other odorants might serve as key odorants at specific concentrations. However, not all odorants are key odorants. The intensity of the odor background influences the attractiveness of the key odorants. Increased complexity is only more attractive in a concentration-dependent range for single compounds in a blend. Orco is necessary to discriminate between two similarly attractive odorants when offered as single odorants and in food odor blends, supporting the importance of single odorant recognition in odor blends. These data strongly indicate that flies use more than one strategy to navigate to a food odor source, depending on the availability of key odorants in the odor blend and the alternative odor offered.

## Introduction

Like other insects, *Drosophilidae* *Drosophila melanogaster* *meigen* *1830* searches for food using the olfactory system. In natural environments, a putative food source such as a fruit emits multiple volatile compounds with varying concentrations and compositions. For example, the smell of an apple contains more than 100 different volatile compounds (Ferreira et al., 2009). This bouquet changes in composition and complexity during ripening and is characteristic of specific maturity states (Mehinagic et al., 2006). Microorganisms contribute their own specific smells to the fruit odor blend. Yeast settles on the surface of the fruit and emits high levels of ethyl acetate (EtOAc) and—as a byproduct of anaerobic metabolism of the fruit sugars—increasing concentrations of ethanol (Alves et al., 2015). Acetobacter sp. converts ethanol into acetic acid (AA; Rao and Stokes, 1953). The smell of ethanol is characteristic of a fermenting apple (Zhu et al., 2003). Ethanol appears to have a specific biological significance for the flies; flies are more attracted to ethanol-containing food odors than odors without ethanol (Schneider et al., 2012). At the food source, they prefer to feed on ethanol-enriched food possibly due to its caloric value (Pohl et al., 2012) and/or intoxicating effect (Devineni and Heberlein, 2013). In addition to food intake, ethanol-enriched food sources are also preferred as oviposition sites (Eisses, 1997; Azanchi et al., 2013). The attraction of flies to ethanol-containing food odors raises the question of whether the smell of ethanol might serve as a key odorant that attracts *Drosophila melanogaster* to a fermenting food source.

The olfactory system of the fly detects and discriminates between different odor blends. Defining the significance of odor information for the animal requires the linkage of olfactory input with internal requirements. Depending on environmental cues and the internal condition, the fly responds with approach, avoidance or indifference. An observed indifference might reflect the meaninglessness of the odor information or a balance between information from two conflicting odors. Although a single odor-specific receptor is expressed in one olfactory receptor neuron (ORN), complex odor blends are most likely not detected based on every single odorant identity. ORNs housing odor specific receptors respond to multiple ligands, and most single odor molecules activate multiple receptor neurons (Wilson, 2013). Therefore, the question arises as to how animals differentiate between two similar food odor blends.

At least three mechanisms might allow the fly to distinguish between similarly complex food odor blends. First, the behavioral response to a food odor is elicited by a key odorant within the blend. Here a key odorant is defined as an odorant that elicits a behavioral difference in a comparative situation. In *Drosophila melanogaster*, odorants that might function as key odorants are compounds of specific biological significance to the fly (Joseph and Carlson, 2015). Second, the complexity of the odor mixture influences its attractiveness. Single odorant compounds from overripe mangos, such as ethanol, AA and phenylethyl acetate, are less attractive than a blend of all three compounds (Zhu et al., 2003). When given the choice between single odorants and a mango-apple food odor blend, flies are more attracted to the complex food odor (Schneider et al., 2012). Third, the fly senses the valences of the single odorants within the blend and evaluates how attractive an odor blend is by adding the valences (Thoma et al., 2014). For example, a walking fly is more attracted to a binary odorant mixture of two similarly attractive odorants than to each odorant separately (Thoma et al., 2014).

First, we investigated whether ethanol and other odorants serve as putative key odorants in food odor blends. By analyzing the behavioral response of *Orco* mutants, we determined whether the odorant is recognized based on an Orco dependent mechanism. Second, we addressed whether an increase in odor blend complexity is always more attractive to the flies. We determined the valence of single odorants and analyzed whether combinations of these single odorants are more attractive than the single odorant itself. Finally, we analyzed the effect of a complex odor background on single-odorant attraction. We show that the attractiveness of an odor blend depends on the presence of key odorants and single compound concentrations rather than increased complexity. For differentiation between two similarly complex food odors, key odorants are required, since Orco mutants fail to distinguish between similarly complex food odors. The presence of key odorants correlates with the biological significance of the compound. Finally, the concentration of the odor background changes the attractiveness of the key odorant.

## Materials and Methods

### *Drosophila melanogaster* Stocks

Flies were raised on ethanol-free standard cornmeal/molasses/yeast/agar medium at 25°C and 60% relative humidity under a 12 h light/12 h dark cycle. The following lines were used: *w*^1118^; *Orco*^1^ (Larsson et al., 2004); w*; *Orco-GAL4* [Bl #26818] and w*; *UAS-Orco* [Bl#23145] from the Bloomington *Drosophila* Stock Center. All strains were backcrossed to *w*^1118^ (Scholz lab) for at least five generations.

### Odor Compounds and Blends

The following odorants were used: ethanol (EtOH; VWR, Germany #20821.321), EtOAc (AppliChem, #A0681), AA (VWR, Germany #20104.298) and acetophenone (AP; SIGMA-ALDRICH, #00790). For every experiment with single odorants, odorants were freshly mixed with distilled water at room temperature. Apple-mango juice (Alnatura, Germany GTIN: 4104420071841) was used as the food odor. Various concentrations of single compounds were mixed with the juice. 0.001% Tween-20 was added to EtOAc and AP solutions to reduce surface tension. When Tween-20 was used, it was added to both odor traps.

### Odor/Odorants Attraction Assay

To determine the attractiveness of an odor, a binary choice trap assay was used (Ogueta et al., 2010). Briefly, 50–80 1-to-3-day-old male flies were collected under CO_2_ sedation in less than 5 min and kept at 25°C for 2 days for recovery. The 3-to-5-day-old flies were introduced into a beaker containing two odor traps at 25°C and 60% relative humidity. After 16 h, the number of flies in the odor traps was determined. A preference index (PI) was only calculated when more than 95% of all tested flies made a decision and entered a trap, otherwise the experiment was discarded. A PI was calculated as follows: PI = (#_A_ – #_B_)/total number of flies that entered odor traps, where #_A_ and #_B_ indicate the fly numbers in trap A and B, respectively. The letter *N* indicates the number of different fly groups tested. PI values differ between 1 and – 1; a positive PI indicates attraction, whereas a negative value indicates aversion.

### Statistical Analysis

Significant differences between two normally distributed data were determined with Student’s* t*-test. For more than two groups, the ANOVA combined *post hoc* Tukey’s honest significant difference (HSD) test was used. The one-sample sign test was performed to test whether attraction differed from random choice. The values are reported as the means ± SEM. Statistical analysis was performed using STATISTICA (Version 7.1)^1^.

## Results

**Figure 1:**
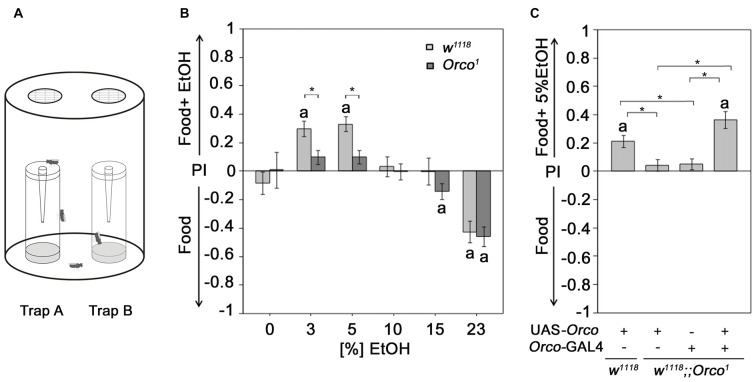
Orco requirement for attraction to EtOH enriched food odor. **(A)** Flies are allowed to choose for 16 h inside the olfactory binary choice trap assay consisting of two odor traps. The attractiveness is determined by the fly number in one trap minus the fly number in the other trap compared to the total number of flies that entered odor traps. **(B)** The control and *Orco*^1^ mutant flies were tested for their attraction to EtOH-enriched food odors with different concentrations (0%–23%) compared to food odor (*N* = 17–41). The stars indicate significant differences between *w*^1118^ and *Orco*^1^ as determined by Student’s *t*-test (*P* < 0.05). **(C)** The loss of EtOH attraction in *Orco*^1^ mutants was restored by Orco transgene expression in an *Orco*-Gal4 dependent manner (*N* = 28–43). The letter **a** indicates significant differences from random choice as determined by the one-sample sign test (*P* < 0.05); here the stars indicate significant differences as determined by ANOVA *post hoc* Tukey’s honest significant difference (HSD) analysis (*P* < 0.05).

To analyze the significance of naturally occurring odors for the behavioral response of flies, we used a binary choice assay consisting of two odor traps. When given a choice between food odors and EtOH-enriched food odors, flies are more attracted to EtOH-containing food odor in a dose-dependent manner (Ogueta et al., 2010; Schneider et al., 2012; Figures 1A,B). They prefer low naturally occurring EtOH concentrations and avoid high EtOH concentrations.

### Orco-Dependent Attraction to EtOH-Enriched Food Odors

Ethanol-enriched food sources are preferred for their caloric value (Pohl et al., 2012) and/or intoxicating effect (Kaun et al., 2011). To ensure that the analyzed behavioral response, i.e., the attraction to an odor, is indeed due to the response of an olfactory stimulus and not the motivation to obtain calories or to be intoxicated, we used *Orco*^1^ mutants with altered odor perception. Orco interacts with the odor-specific receptor and facilitates the functional expression of odor-specific receptors in the olfactory sensory neurons (OSNs) required for single-odor detection (Larsson et al., 2004; Benton et al., 2006). *Orco*^1^ mutants fail to show attraction to 5% EtOH-containing food odors (Schneider et al., 2012). Here, we extended the analysis to concentrations ranging from 3% to 23% EtOH (Figure 1B). As expected, in contrast to control flies, *Orco*^1^ mutants are not attracted to low ethanol concentrations (3%–5% EtOH). At high EtOH concentrations, *Orco*^1^ and control flies show a similar degree of aversion. Our results is consistent with previous findings that olfactory attraction to EtOH is concentration-dependent (Ogueta et al., 2010) and suggest that attraction to low EtOH levels is mediated by an Orco-dependent mechanism (Schneider et al., 2012). Aversion to high EtOH concentrations is Orco-independent. To confirm that the loss of attraction is indeed due to loss of Orco function in OSNs, we restored Orco function in *Orco*^1^ mutants using the *Orco*-Gal4 driver (Larsson et al., 2004; Figure 1C). Expression of *Orco* in the OSNs restores the loss of EtOH attraction in *Orco*^1^ mutants. Thus, the detection of EtOH within a food odor blend, resulting in attraction, requires Orco function in the OSNs.

### Key Odorant Function at Low Odor Concentrations

**Figure 2:**
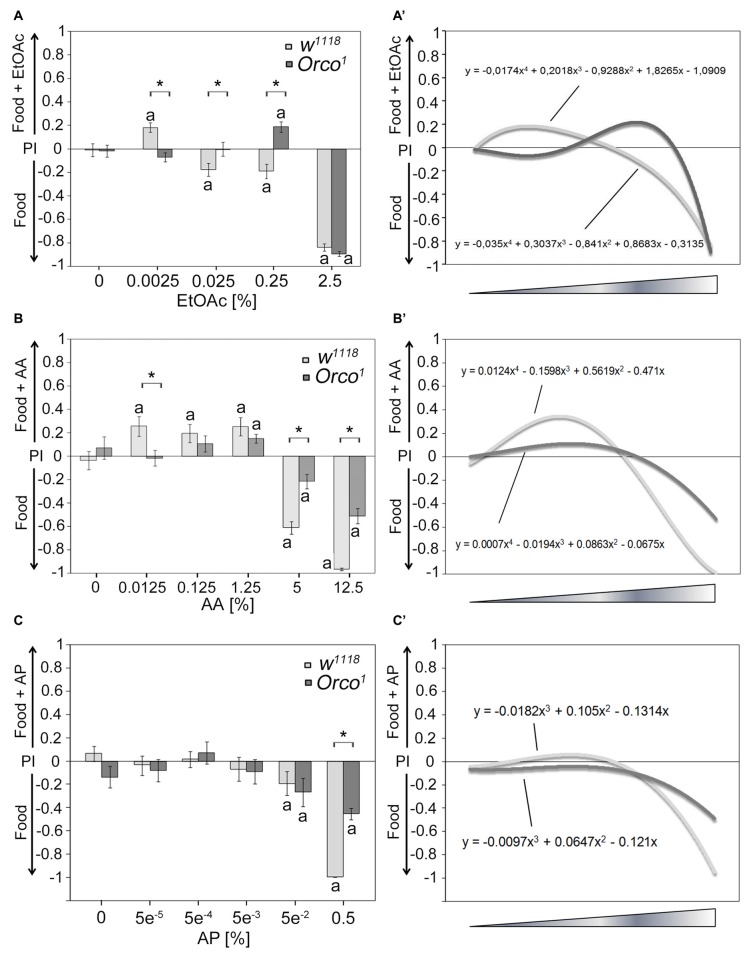
Dose-dependent attraction to ethyl acetate (EtOAc)-, acetic acid (AA)- and acetophenone (AP)-containing food odors. Dose-response curve for different **(A)** EtOAc, **(B)** AA and **(C)** AP concentrations in food odors compared to food odors for control flies and *Orco*^1^ mutants for EtOAc (*N* = 17–48; for AA: *N* = 13–22; for AP *N* = 16–27, respectively). **(A′–C′)** A polynomial analysis of the data presented in **(A–C)** of *w*^1118^ (light gray line) and *Orco*^1^ flies (dark gray line) reveals differences in the kinetics of odor attraction for **(A′)** EtOAc, **(B′)** AA and **(C′)** AP. The letter **a** marks differences from random choice as determined by a one-sample sign test (*P* < 0.05). The stars indicate significant differences as determined by Student’s *t*-test (*P* < 0.05).

Next, we tested whether other odorants might serve as a key odorant among food odors (Figure 2). We used different classes of naturally occurring odorants, including EtOAc, AA and aromatic AP, at different concentrations. Control flies show concentration-dependent attraction to EtOAc- and AA-containing food odors (Figures 2A,B) but fail to show attraction to AP-containing food odors (Figure 2C). High concentrations of EtOAc-, AA- and AP-containing food odors are aversive (Figures 2A–C). To test whether Orco is also required for single odor recognition within a food odor blend, we next examined whether *Orco*^1^ mutants distinguish between a food odor and a food odor with different concentrations of EtOAc, AA and AP (Figure 2; Supplementary Figure S1). *Orco*^1^ mutants are still attracted to EtOAC or AA in food odor mixtures; however, the threshold to respond with attraction shifts to a higher concentration. Control *Orco*^1^ mutants do not show attraction to AP-containing food odors. In *Orco*^1^ mutants, higher EtOAc, AA and AP concentrations are less aversive (Figures 2A–C). To obtain better resolution of Orco function in odorant detection, we applied a polynomial analysis to the data (Figures 2A′–C′). The analysis supports a shift of attraction to higher concentrations for EtOAc and a reduced sensitivity for AA (Figures 2A′,B′) and indicates that Orco enhances sensitivity to detect EtOAc and AA in complex food odor blends. *Orco*^1^ mutant flies do not show a shift in attractiveness to higher concentrations of AP (Figure 2C′) and are less averse to high concentrations of AP in food odors. Normally, flies do not respond with attraction to increasing concentrations of AP. Orco enhances the detection of aversive AP concentrations.

### Orco Independent Mechanism of Single Odorant Detection

**Figure 3:**
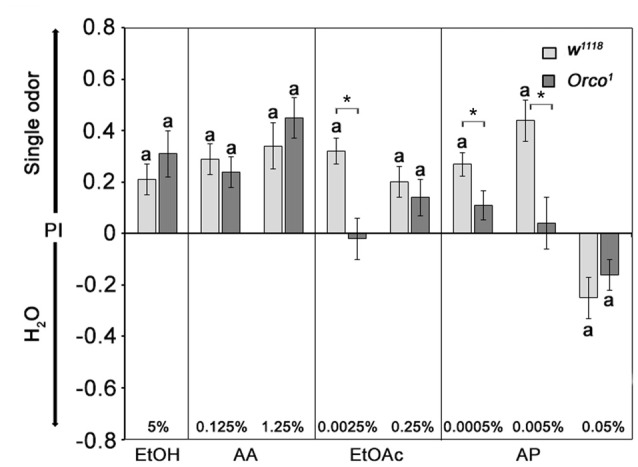
Attraction to single odorants in *Orco*^1^ mutants. The *w*^1118^ flies are more attracted to single odorants than water. *Orco*^1^ mutants behave similar to controls for most tested odorants, except for 0.0025% EtOAc and 0.005% AP, where no attraction was observed (*N* = 13–37). Control flies and mutants show aversion to 0.05% AP (*N* = 26, 22). Significant differences from random choice was determined by the one-sample sign test and are indicated by the letter **a** (*P* < 0.05), whereas the stars indicate differences between control and mutant flies at the same concentration as determined by the Student’s *t*-test (*P* < 0.05).

The observed attraction to EtOAc and AA in *Orco*^1^ mutants suggests that *Orco*^1^ mutants still sense these odors based on an Orco-independent mechanism. To investigate whether an Orco-independent mechanism exists in EtOAc and AA detection, we investigated whether *Orco*^1^ mutants still recognized the single odorants of EtOAc and AA independent of the food odor context. Flies chose between a single odorant of EtOAc, AA, EtOH or AP and humidified air at similar concentrations that elicit attraction in food odors (Figure 3). Control flies are more attracted to EtOH, AA and EtOAc at all tested concentrations and to AP at 0.0005% and 0.005%. Flies show aversion to 0.05% AP. A similar concentration of AP was also aversive when present in food odor blends (see Figures 2C, 3). In contrast to controls, *Orco*^1^ mutants are not attracted to 0.0025% EtOAc, consistent with the idea that the detection of low concentrations of EtOAc in food odors is due to an Orco-dependent mechanism. At higher EtOAc concentrations *Orco*^1^ mutants show attraction to EtOAc, suggesting that the detection of higher EtOAc is Orco-independent. *Orco*^1^ mutants are still attracted to EtOH, AA and AP at low concentrations. Similar concentrations do not result in attraction when presented in food odor blends (Figure 3). These results indicate that attraction to EtOH, AA and AP is not only based on Orco dependent mechanism.

### Relative Attractiveness of Single Odorants

**Figure 4:**
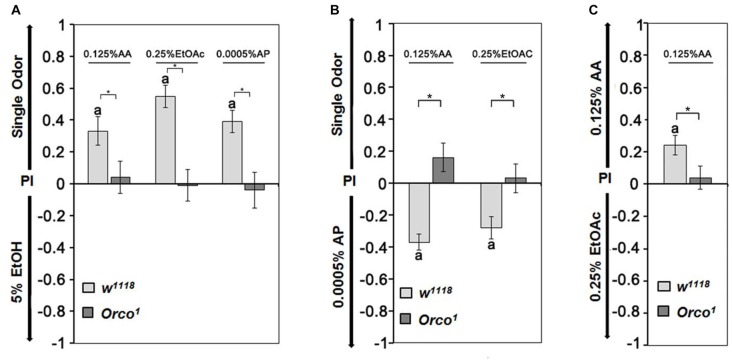
Relative attractiveness of single odorants. **(A)** Control flies are more attracted to 0.125% AP, 0.25% EtOAc and 0.0005% AP than 5% EtOH, but there are no observed differences between the odorant pairs for *Orco*^1^ mutants (*N* = 12–18). **(B)** Compared with 0.0005% AP, flies are averse to AA and EtOH, whereas *Orco*^1^ flies are indifferent (*N* = 16–27). **(C)** Flies prefer AA over EtOAc, and both odorants are equally attractive for *Orco*^1^ mutants (*N* = 20, 25). A significant difference from random choice is labeled with the letter **a** as determined by the one-sample sign test (*P* < 0.05) and differences between controls and *Orco*^1^ flies are labeled with stars as determined by Student’s *t*-test (*P* < 0.05).

To further investigate whether the identity of single odorants influences the choice of a fly when two similar attractive odorants are offered, we first determined whether flies distinguish between two similar attractive odorants (Figure 4). In the choice situation, we used EtOH, AA, EtOAc and AP at concentrations that elicited similar preference indices ranging from 0.2 to 0.3 when offered in comparison to humidified air (Figure 3). Control flies are more attracted to AA, EtOAc and AP than 5% EtOH (Figure 4A). They show a higher attraction to 0.0005% AP than AA or EtOAc (Figure 4B) and a higher attraction to 0.125% AA than to 0.25% EtOAc (Figure 4C). When comparing the single odorants combinations with each other, the attractiveness for different odorants can be ranked. For control flies, AP is the most attractive, and EtOH is the least attractive odorant (0.0005% AP > 0.125% AA > 0.25% EtOAC > 5% EtOH). The preferential attraction for any tested odorant pair was missing in *Orco*^1^ mutants (Figures 4A–C). Therefore the comparison between two odorants depends on the recognition of an Orco dependent mechanism and the valence of the single odorants changes compared to the alternative.

### Influence of Odor Complexity on Attractiveness

We have shown that in a choice situation 5% EtOH containing food odors are more attractive than food odors without EtOH (Figure 1). The question arise whether 5% EtOH is detected based on its identity within the food odor blend or increase of complexity in the food odor blend. Normally flies prefer a food odor blend over 5% EtOH. This attraction increases when 5% EtOH is added to the food odor and this increase is not observed in *Orco*^1^ mutants although these mutants still prefer food odors over 5% EtOH (Schneider et al., 2012). These observations indicate that flies normally detect 5% EtOH in food odor blend based on their identity or alternatively due to an increase in odor complexity.

**Figure 5:**
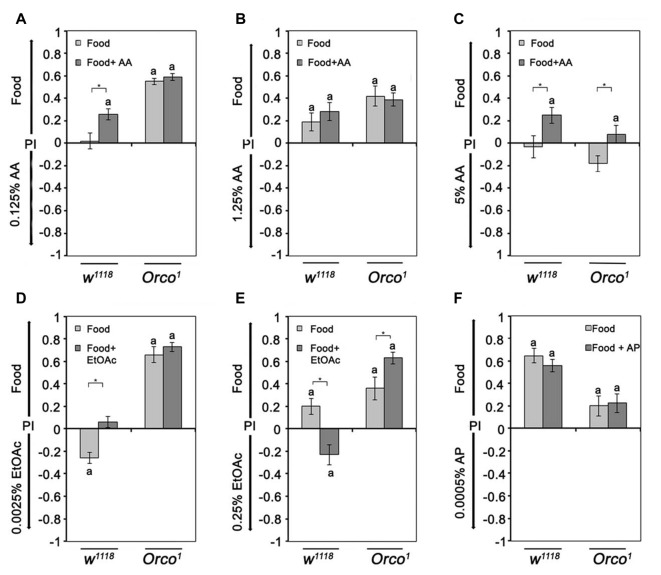
Sensing of differences between similar complex food odors.** (A–F)** The attraction to food odor or food odors with a specific single odorant vs. a single odorant was compared in control and *Orco*^1^ flies. The tested AA concentrations were, in **(A)**, 0.125%, in **(B)**, 1.25% and in **(C)** 5% (*N* = 14–23). For EtOAc, 0.00025% and 0.25% were tested in **(D,E)**, respectively. **(F)** For comparison of AP and food odor with or without AP, 0.0005% AP was used (*N* = 16–25). Bars labeled with **a** are significantly different from random choice as determined by the one-sample sign test, and stars indicate significant differences between control and *Orco*^1^ flies for a specific choice situation according to Student’s *t*-test (*P* < 0.05).

To investigate the influence of odor complexity on the attractiveness of two different odors, flies were offered a choice between different concentrations of AA or EtOAc and food odors. We observed three classes of responses. First, flies are more attracted to food odors when comparing 1.25% AA or 0.25% EtOAc to food, consistent with the expectation that more complex odor blends are more attractive. Second, flies are indifferent when the food odors is offered in comparison to a single odorant like 0.125% AA. Third, a single odorant is more attractive than the food odor (Figure 5D). Control flies prefer 0.0025% EtOAc over food odor, but find 0.25% less attractive than food (Figures 5D,E). The last class of responses shows that a single odorant can be more attractive than a complex odor blend, and the attractiveness of the single odorant in a choice situation depends on the single odorant concentration.

To further investigate the effect of increased complexity, we added the same concentration of the single odorant to the food odor (Figure 5). An increase in complexity should result in an increase in attractiveness in comparison to the choice situation if the single odorant is not contained in the food odor. Again, we find three classes of behavioral responses. In the first class, adding the odorant to the food odor blend increases the attraction to the enriched food odor in comparison to the single odorant (0.125% AA; Figure 5A). The second class is indifferent to an increase (1.25% AA and 0.0005% AP; Figures 5B,F). In the third class, the attractiveness of the food odor is reverted into aversion (0.25% EtOAC; Figure 5E). The second and third classes of behavioral responses indicate that more complex odor blends are not necessarily more attractive and that the attraction depends on the concentrations of single odorants within the blend.

To analyze how much this behavioral choice is influenced by the identity of the odorants, we analyzed *Orco*^1^ mutants in similar experiments. *Orco*^1^ mutants prefer the food odor over the single odorants in all but one case (5% AA; Figure 5B). An increase in complexity by adding a single odorants to the food odor did not increase the attractiveness to food odors in four of the six tested conditions. In part, these results are consistent with the fact that in *Orco*^1^ mutants, the identity of a single odor cannot be sensed due to the loss of the odor specific receptor. However, the loss of Orco-dependent specific odorants recognition cannot account for the observed increased attractiveness when higher concentrations of EtOAc were added to the food odor (5% AA and 0.25% EtOAc; Figure 5E). Adding 0.25% EtOAc to food odors in comparison to EtOAc shifts the attractiveness of *Orco*^1^ mutants to increased attraction to the EtOAc-enriched food odors (Figure 5E). These results indicate that an Orco independent mechanism mediates odor attraction in addition to an Orco dependent mechanism at low odorants concentrations.

### Influence of Odor Background Intensity on Single-Odorants Attraction

**Figure 6:**
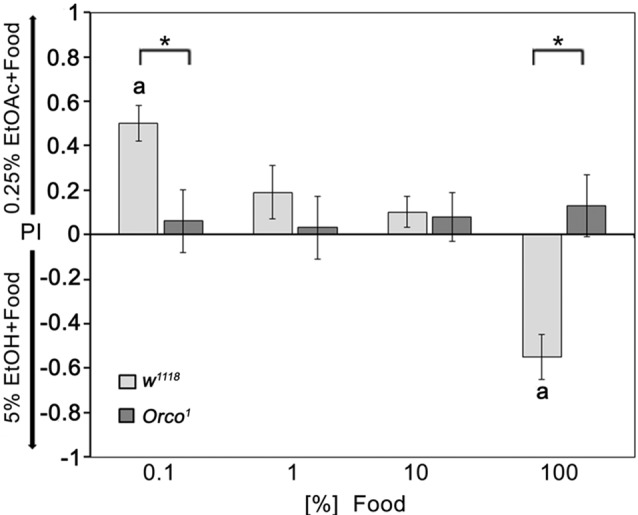
Influence of background food odor concentration on single odorant discrimination. The attraction to EtOH-enriched food odor dependence on food odor concentration compared to EtOAc-enriched food odors in control flies. In these different choice pairs, the concentrations of 5% EtOH and 0.25% EtOAc were not changed. The food odors were diluted up to 1000-fold with water (0.1% food); (*N* = 11–21). *Orco*^1^ mutant flies do not prefer either odor mixture of the two odor pairs offered to the flies (*N* = 10–20). Bars labeled with **a** are significantly different from random choice as determined by a one-sample sign test (*P* < 0.05) and the stars indicate significant difference between control and *Orco*^1^ mutant flies using the Student’s *t*-test (*P* < 0.05).

To address how food odor influences the behavioral response to individual odorants, we offered flies a choice between a fixed concentration of 0.25% EtOAc and 5% EtOH diluted with different concentrations of food odors (Figure 6). Normally, flies prefer 0.25% EtOAc over 5% EtOH, which was still the case when both odorants were presented in a strongly diluted food odor background. After increasing the food odor concentration, flies reduce their attraction to 0.25% EtOAc and increase their attraction to ethanol containing food odors. *Orco*^1^ mutants do not change their indifference toward both odor sources. Thus, single odor discrimination depends on the intensity of the odor background; at a certain threshold, the food odor influences the behavioral response to the single odorants and might change the attractiveness of the single odorants. The discrimination between two similarly complex food odors that differ in only one odorants depends on Orco function. In both odor traps, the concentration of food odors increases equally; the composition of the food odor does not change, but the intensity of additional odor compounds that influence the behavioral outcome do.

### Influence of Additional Odorants on Single-Odorant Attraction

**Figure 7:**
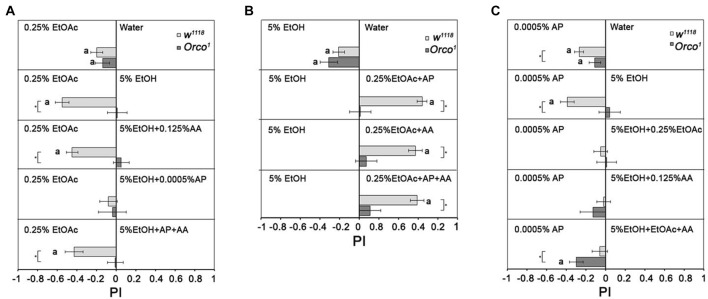
Influence of odor composition on individual odorant discrimination. Behavioral response of control and *Orco*^1^ flies to single odorants compared to other single odorants or binary and tertiary combinations with 0.0005% AP and 0.125 AA are shown. In **(A)**, 0.25% EtOAc is compared to 5% EtOH with various odor combinations (*N* = 12–27); in **(B)**, 5% EtOH and 0.25% EtOAc with various odor combinations (*N* = 12–28) and in **(C)**, 0.0005% AP and 5% EtOH various odor combinations (*N* = 14–37). Significant difference from random choices are labeled with the letter **a** as determined by the one-sample sign test; (*P* < 0.05) and differences between *control* and *Orco*^1^ flies are labeled with a star as determined by Student *t*-test (*P* < 0.05).

To analyze how additional odorants influence the attractiveness of EtOH in comparison to 0.25% EtOAc and to eliminate the influence of food odor detection by Orco independent mechanisms, we added different single odorants to EtOH (Figure 7A). As shown in Figures 3, 4A, EtOAc is more attractive than water or 5% EtOH. Adding AA to 5% EtOH does not change the attractiveness of control flies to 0.25% EtOAc. However, adding 0.0005% AP to 5% EtOH results in indifference to both odor sources (Figure 7A). This indifference changes again to attraction for EtOAc when 0.125% AA is added to the binary mixture. *Orco*^1^ mutants choose all odors equally, suggesting that differentiation between odorant combinations depends on the identity of the single odorant compounds. To analyze whether the attractiveness of 0.25% EtOAc changes in comparison to 5% EtOH when additional odorants are added to EtOAc, flies were given the choice between EtOH and 0.25% EtOAc supplemented with different single odorants (Figure 7B). EtOH does not become more attractive in any of the tested combinations. Thus, the increase in odor complexity of the blends containing EtOH does not make the blend more attractive than the single odorants. Again, the attractiveness of odors was evaluated based on an Orco dependent mechanism, since *Orco* mutants are similarly attracted to both odors in the combination.

To investigate whether 5% EtOH-containing binary and tertiary odorant mixtures are also less attractive when odorants other than EtOAc are presented, we offered different odorant combinations with 5% EtOH in comparison to 0.0005% AP (Figure 7C). Flies are equally attracted to binary EtOH-containing mixtures as to 0.0005% AP. Adding a third odorant to the binary EtOH-containing odor blend does not change this balance. This result again suggests that an increase in complexity does not necessarily correlate with increased attractiveness. *Orco*^1^ mutants are more attracted to AP than to EtOH-containing tertiary mixtures.

**Figure 8:**
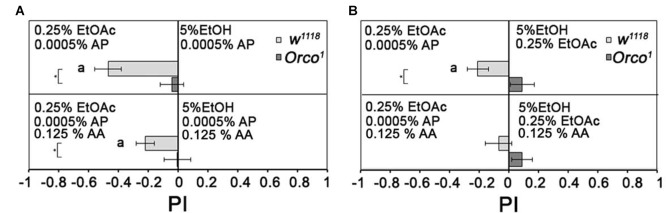
Comparison of binary and tertiary odor mixtures. Attraction to different binary and tertiary odor mixtures of 5% EtOH, 0, 25 EtOAc, 0.0005% AP and 0.125% AA were analyzed for control and *Orco*^1^ mutants in **(A)** between 0.25% EtOAc and 5% EtOH and in **(B)** for 0.0005% AP and 5% EtOH (*N* = 14–29). Significant differences from random choice are labeled with the letter **a** as determined by the one-sample sign test; *P* < 0.05 and differences between control and *Orco*^1^ flies are labeled with a star as determined by Student’s *t*-test (*P* < 0.05).

To analyze whether flies normally differentiate between binary and tertiary odorant blends, we added one or two additional odorant to 0.25% EtOAc and/or 5% EtOH (Figure 8). Flies are still more attracted to a binary mixture containing 0.25% EtOAc than to binary mixtures containing 5% EtOH. They were also more attracted to a tertiary mixture containing 0.25% EtOAc than to tertiary mixtures containing 5% EtOH, but to a lesser extent (Figure 8A). When both tertiary mixtures contain EtOAc, flies are equally attracted to both odorant mixtures (Figure 8B). *Orco*^1^ mutants did not distinguish between all odorant combinations. Therefore, flies still distinguish between two tertiary odorant mixtures likely due to the identity of single odorant compounds.

## Discussion

Insects such as *Drosophila melanogaster* use sensory information derived from volatile odorants as important clues to search for and select appropriate food sources (Wilson and Mainen, 2006; Chow and Frye, 2009). The olfactory input derived from a food source consists of a complex odor bouquet. We provide evidence that flies recognize specific odorants within the food odor bouquet based on their identity and that the concentration of these odorants determines the attractiveness of the food odor blend. The intensity of the odor background might change the evaluation of attractiveness of the single key odorants.

### Evidence for Key Odorants that Elicit Attraction within Food Odors

Humans, with a structurally analogous olfactory system, fail to discriminate between single odorant compounds when more than three or four compounds are present within the blend (Laing and Francis, 1989; Livermore and Laing, 1996). A food source such as an apple emits over 100 different odorant compounds (Ferreira et al., 2009). Therefore, the likelihood that an insect evaluates every single odorant compound is rather low. Key odorants for biologically significant information facilitate decision making and ultimately the behavioral response to the odor. Therefore, the odorant must be recognized based on its identity within an odor blend. We found that ethanol, EtOAc and AA within food odors were attractive in a concentration-dependent manner (Figure 1). These results are consistent with the common characteristic of a behavioral response curve for increasing concentrations of single odorants. In *Drosophila*, low odorant concentrations are attractive, and increasing odorant concentrations become less attractive and eventually aversive (Wang et al., 2003; Semmelhack and Wang, 2009). Flies recognize ethanol at concentrations that results in attraction within a food odor based on its identity, since *Orco* mutants fail to show attraction at any tested concentration of ethanol (Figure 1). Given that Orco is required for odor-specific receptor interaction in ORNs (Larsson et al., 2004; Benton et al., 2006), the results indicate that Orco interacts with an ethanol specific olfactory receptor. A possible candidate for this Orco-interacting receptor is the odor-binding protein Lush, which binds ethanol at physiological relevant concentrations (Thode et al., 2008).

Unlike ethanol, flies only detect EtOAc and AA at low concentrations in an Orco-dependent manner within a food odor blend (Figure 2). At higher odorant concentrations, *Orco* mutants are still attracted to EtOAc- and AA-containing food odors. This detection could be due to at least four different mechanisms. First, Orco enhances the detection of single odorants within a blend. It has been proposed that Orco enhances the sensitivity of the OR response to odorants over a wider range of concentrations in flies and moths (Getahun et al., 2013; Nolte et al., 2013). Second, an Orco-independent mechanism regulates the attraction to a higher concentration of EtOAc and AA that is normally suppressed by the Orco dependent mechanism. In *Drosophila* larvae, the behavioral response to EtOAc is mediated over a concentration range by two different receptors. The Or42a receptor responds to high concentrations of EtOAc, whereas Or42b responds to low concentrations (Kreher et al., 2008). A similar system might exist in adult flies, and both receptors might interact with Orco differently. Third, other chemical properties associated with the odorants are evaluated as attractive. Evidence for an Orco-independent mechanism for odorant detection regulating attractiveness comes for example from the observation that *Orco* mutants still differentiate between different concentrations of ethanol and AA and high concentrations of EtOAc and water (Figure 3). The Orco independent mechanism might be due to the function of ionotropic glutamate receptors that have been shown to be important for the regulation of the chemosensory response (Benton et al., 2009). Different chemical properties are also sensed by the antenna, the structure housing the ORNs. Flies sense differences in humidity with a hygrosensing system localized to specialized sensory hairs on the antenna (Liu et al., 2007) and sense differences in acidity with the ionotropic receptor IR64a expressed in OSNs (Ai et al., 2010). Finally compounds within the food odor might interfere with the perception of higher concentration of EtOAc and AA. The concentration of the food odor background can change the attraction to similar key odorant concentrations within the food odor (Figure 6).

In contrast to ethanol and low EtOAc or AA concentration, different AP concentrations within a food odor do not elicit attraction (Figure 2), although 0.0005% AP has the highest valence as a single odorant (Figure 4). It is possible that AP is already present in mango blends at concentrations optimal for attraction. If this was the case in all combinations where food odor and a single odor are offered, flies should be more attracted to the food odor, which was not the case when EtOAc and food odors were presented (Figure 3). In addition, AP is found only in very low amounts in mango blends (less than 0.01 mg/kg; Pino et al., 2005). Thus AP does not function as a key odorant in food odor blends for *Drosophila melanogaster*. However, ethanol is a key odorant in food odor blends at all concentrations that elicit attraction, and EtOAc and AA are only key odorants at specific concentrations.

### The Attractiveness of an Odorant Depends on the Alternative

Normally, flies discriminate between two odorant cues by interplay between spontaneous and experience-dependent processes (Hallem et al., 2004; Xia and Tully, 2007; Parnas et al., 2013). The valence of the odorant is determined by the behavioral response, which does not necessarily represent a fixed quality of the odorant but is the motivational significance of the odorant (Ai et al., 2010; Min et al., 2013). Consistent with this, we found that naïve flies discriminate between two odorants with similar valence and choose one over the other. The attraction of two odorants with similar valence is relative and depends on the alternative offered (Figure 4). The valences of the odorants can be ranked, with 0.0005% AP as the most attractive single odorant and 5% EtOH the least. Male flies prefer 0.125% AA over 5% EtOH, similar to *Drosophila melanogaster* females that prefer the AA-enriched oviposition site over EtOH-enriched oviposition sites (Eisses, 1997). Evidence that the valence of a single odorant depends on the alternative is also seen in the case of AP. Normally, AP is the most attractive single odorant (Figure 4). In combination with 5% EtOH, AP becomes less attractive than the single odorant of 0.25% EtOAc (Figure 7A).

Innate odor responses are flexible, as the change in odor contexts in the external environment must be evaluated against the internal state and requirements of the animal (Riffell, 2012; Su and Wang, 2014). The valences of 0.25% EtOAc and 5% EtOH change when food odorants are present. In a context of low food odors, 0.25% EtOAc is more attractive than 5% EtOH; when the food odor intensifies, 5% EtOH becomes more attractive (Figure 6). High levels of EtOAc are provided by yeast (a putative protein source), whereas EtOH is generated from carbohydrates provided by the fruit (Alves et al., 2015). An increase in food odor concentration might signal the distance to a food source. To navigate to the food source flies must identify whether the source is suitable for the nutritional requirements and/or as an oviposition/mating site. Therefore, the odor context influences the significance of the single odorant for the response of the fly.

The changes in valence for 0.25% EtOAc and 5% EtOH when the food odor background increases show that the intensity of the food odor background suppresses the attraction to EtOAc and/or enhances the attraction to 5% EtOH. A similar phenomenon has been reported for CO_2._ Here, the avoidance of CO_2_ can be suppressed by activating different inhibitory pathways in the neural system for fruity odors (Jones et al., 2007; Turner and Ray, 2009; Su and Wang, 2014). Since in both sites of the choice assay the food odor background increased equally, the presence of increased intensity of food odor background has different effects on the attractiveness of EtOAc and EtOH.

### Influence of Complexity on Attractiveness of Odor Blends

Single odorant components are less attractive than the blend of all three compounds (Zhu et al., 2003). Therefore, increased odor complexity might be, *per se*, more attractive than single odorants. However, we found that this was not true for food odors enriched with 0.0025% EtOAc. Here, the single odorant EtOAc was more attractive than the more complex food odor blend (Figure 5D). Adding 0.25% EtOAc to food odors does not make them more attractive (Figure 5E) as predicted if an increase in complexity alone makes odors more attractive. Interestingly, in *Orco*^1^ mutants, the degree of attractiveness for food odors varied from 0.2 to 0.8 PIs depending on the offered single-odorant alternative. If complexity was the only factor influencing attractiveness, food odors should be preferred in all combinations to a similar extent.

Therefore, the attractiveness of odor blends containing key odorants do not depend on fixed valence values for single odorants. The valences of single key odorants depend on the alternatives and the intensity of the odor background. Finally, complex odor blends are not necessarily more attractive. The behavioral response of *Drosophila melanogaster* to a food odor blend is driven by only a few odorant compounds with biological significance. These results support previous findings in other insects, such as moths, where only a few compounds within a blend are important for insects to navigate in a spatially and intermittent complex odor and a turbulent odor plume (Riffell, 2012).

## Ethics Statement

This study does not involved human subjects and was conducted in accordance to the guidelines of the Deutsche Forschungsgemeinschaft (DFG).

## Author Contributions

TG and JH contributed equally to the concepts of the work, acquired and analyzed data. SB contributed to the concept of the work, acquired data and drafted the work. HS contributed substantially to the conception and design of the work, the interpretation of the data, wrote the manuscript together with TG, JH. All authors approved the version to be published and agreed to be accountable for all aspects of the work.

## Conflict of Interest Statement

The authors declare that the research was conducted in the absence of any commercial or financial relationships that could be construed as a potential conflict of interest.

## Footnotes

^1^www.Statsoft.com
